# Metformin as a Therapeutic Target in Endometrial Cancers

**DOI:** 10.3389/fonc.2018.00341

**Published:** 2018-08-28

**Authors:** Teresa Y. Lee, Ubaldo E. Martinez-Outschoorn, Russell J. Schilder, Christine H. Kim, Scott D. Richard, Norman G. Rosenblum, Jennifer M. Johnson

**Affiliations:** ^1^Department of Medical Oncology, Thomas Jefferson University, Philadelphia, PA, United States; ^2^Department of Obstetrics and Gynecology, Thomas Jefferson University, Philadelphia, PA, United States

**Keywords:** tumor microenvironment, metabolism, metformin, endometrial cancer, reverse Warburg

## Abstract

Endometrial cancer is the most common gynecologic malignancy in developed countries. Its increasing incidence is thought to be related in part to the rise of metabolic syndrome, which has been shown to be a risk factor for the development of hyperestrogenic and hyperinsulinemic states. This has consequently lead to an increase in other hormone-responsive cancers as well e.g., breast and ovarian cancer. The correlation between obesity, hyperglycemia, and endometrial cancer has highlighted the important role of metabolism in cancer establishment and persistence. Tumor-mediated reprogramming of the microenvironment and macroenvironment can range from induction of cytokines and growth factors to stimulation of surrounding stromal cells to produce energy-rich catabolites, fueling the growth, and survival of cancer cells. Such mechanisms raise the prospect of the metabolic microenvironment itself as a viable target for treatment of malignancies. Metformin is a biguanide drug that is a first-line treatment for type 2 diabetes that has beneficial effects on various markers of the metabolic syndrome. Many studies suggest that metformin shows potential as an adjuvant treatment for uterine and other cancers. Here, we review the evidence for metformin as a treatment for cancers of the endometrium. We discuss the available clinical data and the molecular mechanisms by which it may exert its effects, with a focus on how it may alter the tumor microenvironment. The pleiotropic effects of metformin on cellular energy production and usage as well as intercellular and hormone-based interactions make it a promising candidate for reprogramming of the cancer ecosystem. This, along with other treatments aimed at targeting tumor metabolic pathways, may lead to novel treatment strategies for endometrial cancer.

## Endometrial cancer

Cancer of the endometrium is the fifth most common malignancy in women worldwide, with 455,000 new cases diagnosed worldwide in 2015 (1). The incidence is rising, and is noted to be much higher in developed than developing countries (1, 2). The American Cancer Society estimates that 63,230 new cases will be diagnosed in the United States in 2018 (representing 7% of cancer diagnoses in women), with 11,350 predicted deaths (3). The majority of cases arise in the post-menopausal period, but up to 14% of cases occur in women age 40 or younger (4). The principal risk factor for development of endometrial cancer is exposure to endogenous and exogenous estrogens, which is influenced by factors such as age at menarche and menopause, parity, use of unopposed estrogen therapy or other hormonal therapies (e.g., tamoxifen), and a host of metabolic factors including obesity. 5–25% of cases are also associated with high risk germline mutations, particularly those affecting DNA mismatch repair pathways, leading to early onset of disease (5).

The most common classification system divides endometrial cancers into two subtypes (6). Type I cancers are low-grade, diploid, endometrioid, and hormone-receptor positive, carrying a better prognosis. They frequently display mutations in phosphate and tensin homolog (*PTEN*). Type II cancers (which include the serous, clear cell, mixed cell, undifferentiated, and carcinosarcoma histologies) are high grade, non-endometrioid, aneuploid, and hormone-negative, with higher rates of metastasis and worse prognosis. They tend to occur in older patients and are more likely to have mutations in the tumor suppressor p53. Type II cancers make up only 10% of endometrial cancers but account for almost 50% of relapses and deaths (7), suggesting a fundamental biologic difference between the two subsets.

Most cases are diagnosed at an early stage due to the early detection sign of abnormal bleeding. Standard treatment for apparent stage I endometrial cancer consists of surgical resection (primary hysterectomy with bilateral salpingo-oophorectomy with possible lymph node mapping). Disease that is confirmed to be uterine-confined with low risk features, can be treated with surgery only and has a >90% relapse-free survival rate at 5 years (8). Radiation decreases local relapse rates but does not affect relapse at distant sites or increase overall survival (9, 10). Trials of adjuvant chemotherapy alone with cyclophosphamide, doxorubicin, and cisplatin demonstrated no significant improvement in progression-free survival, overall survival, or relapse (11). Combined adjuvant chemoradiation has shown a slight increase in progression-free survival but not overall survival (11).

For metastatic or recurrent disease, management may include surgery or radiation (if localized to a single site); those with unresectable disease may sometimes receive primary chemotherapy followed by cytoreductive surgery. For disease not amenable to local therapy, a carboplatin-paclitaxel combination is increasingly used as a first-line alternative to the traditional cisplatin, paclitaxel, and doxorubicin (7). With respect to hormonal therapy in advanced disease, a 33% response rate was noted after alternating tamoxifen and medroxyprogesterone (11–13). In recurrent or metastatic disease, progestogens, tamoxifen alternated with megestrol, gonadotropin-releasing hormone analogues, selective estrogen receptor modulators, and aromatase inhibitors have been used with response rates ranging from 11 to 56% (13, 14). Ultimately, the response rates for recurrent advanced disease are low, and there are no standard second line therapies (13, 15).

This lack of effective treatment for advanced stage endometrial cancer has led to exploration of alternative therapeutic modalities. In particular, numerous studies have examined the effectiveness of targeted therapies acting on the phosphoinositide 3-Kinase (PI3K)/Protein kinase B (Akt)/mammalian target of rapamycin (mTOR) pathway, epidermal growth factor receptor (EGFR), human epidermal growth factor receptor 2 (HER2), and vascular endothelial growth factor (VEGF), reviewed elsewhere (11). The results of single-agent mTOR inhibitor treatment, or EGFR and HER2 inhibitors have been disappointing, with response rates of 0–12%. Anti-angiogenic drugs such as bevacizumab, sunitinib, brivanib, and lenvatinib have resulted in slightly higher objective response rates of 14–19%. Studies of additional targets, including fibroblast growth factor receptor (FGFR), luteinizing hormone releasing hormone (LHRH), poly ADP-ribose polymerase (PARP), and Programmed Death-1/Programmed Death Ligand-1 (PD-1/PD-L1) are underway. At this point, no targeted therapies have been approved. Therefore, further interest has been focused on other factors that could contribute to development and progression of endometrial cancer.

## Associations with metabolic syndrome, obesity, and metabolism

Among the risk factors associated with endometrial cancer, metabolic syndrome (a constellation of obesity, hyperglycemia, hypertension, and hyperlipidemia) has attracted a large amount of interest in recent years. Multiple associative studies have suggested that the metabolic syndrome is a risk factor for development of many different types of cancers (16–18), including endometrial cancer (19–24). A meta-analysis of 6 studies from North America, Europe, and China estimated a relative risk (RR) for endometrial cancer of 1.89 in patients with metabolic syndrome (95% confidence interval [CI] 1.34–2.67, *p* = 0.001) (25). Another meta-analysis of 7 European cohorts reported a 56% increase in endometrial cancer risk per increase of one standard deviation in a composite metabolic risk score derived from sex- and cohort-specific means in body mass index (BMI), blood pressure, plasma cholesterol, triglycerides, and glucose (18). Apart from incidence, Ni and colleagues reported increased endometrial cancer stage, grade, vascular invasion, tumor size, and lymphatic metastasis in patients with metabolic syndrome, as well as decreased overall survival (26).

The individual components of the metabolic syndrome have also been studied in relation to endometrial cancer risk, but it is unknown if their contribution is additive or synergistic. In particular, obesity has been noted to be strongly associated with risk of endometrial cancer in several case-control studies and meta-analyses (21–25, 27, 28). Multiple measures of adiposity, including BMI, waist circumference, waist-to-hip-ratio, and hip circumference, have been found to be directly associated with endometrial cancer incidence. Increased waist circumference and BMI have also been shown to be significantly associated with increased risk of overall mortality from endometrial cancer (29, 30). Other studies have demonstrated positive albeit less robust association between endometrial cancer and the other components of the metabolic syndrome: hypertension (21–24), hyperlipidemia (21–24), and hyperglycemia or diabetes mellitus (19, 21–25, 31, 32). The association between diabetes and endometrial cancer appears to be partially confounded by co-existing overweight/obesity (33, 34). However, elevated risk of endometrial cancer in patients with diabetes has been reported even after adjustment for BMI, with one meta-analysis including 29 cohort studies reporting a summary relative risk of 1.89 [95% CI, 1.46–2.45, *p* < 0.001] (32). This study also noted a small increased risk of disease-specific mortality in diabetic patients with endometrial cancer (RR 1.32, 95% CI, 1.10–1.60; *p* = 0.003).

The major driver of increased risk of endometrial and other hormone-responsive cancers in obesity is thought to be the generation of a hyper-estrogenic state caused by the presence of the aromatase enzyme in adipose tissue (35). This enzyme catalyzes conversion of androgens to estrogens, making adipose tissue a key source of estrogens in post-menopausal women. In addition, adiposity has been associated with other factors that may drive tumorigenesis in general, including increased inflammation, depressed immune function, and chronic insulin resistance and hyperinsulinemia. Endometrial cancer patients have been shown to have increased markers of insulin resistance, including higher fasting insulin levels and elevated non-fasting and fasting C-peptide levels (36, 37). Supporting this link between abnormal glucose metabolism and cancer risk is the observation that better diabetic control is associated with decreased endometrial cancer risk (21). Ultimately, these data suggest that abnormal metabolism, including insulin resistance and hyperglycemia, may play a role in the development of endometrial cancer and thus represent a possible therapeutic target.

## Metformin repurposing and epidemiologic data from endometrial cancer

In recent years there has been growing interest in drug repurposing or repositioning, a process which seeks to identify new pharmacologic properties (e.g., anti-tumorigenic) of existing medications for use as primary or adjuvant treatments for other conditions (38, 39). These drugs are already well-studied in terms of tolerability and side effects, often inexpensive, and amenable to retrospective and associative studies as many patients are already taking them for other indications. The association between obesity, diabetes, hyperinsulinemia, and endometrial cancer has led to the hypothesis that medications which target glucose metabolism such as metformin may be effective in preventing or treating such malignancies. One drug that has received a significant amount of attention in this arena has been metformin [1,1-dimethylbiguanide] which is a first line oral antihyperglycemic agent used in the treatment of type 2 diabetes (40). Broadly, its effects include lowering of blood glucose concentrations, increasing insulin sensitization, and reducing plasma fasting insulin levels. Furthermore, unlike with some oral hypoglycemic medications and insulin, metformin users show a tendency toward sustained weight loss (41). The low toxicity of metformin makes it especially interesting as a potential adjunctive therapy, or even as monotherapy for patients with contraindications to chemotherapy or considerations such as the desire to preserve fertility.

Many investigators have sought to examine the effect of metformin exposure on the development of endometrial cancer (Table 1). Multiple epidemiologic studies have reported lower overall cancer incidence in metformin users, reviewed by several groups (47–53). Studies evaluating the relationship between metformin use and endometrial cancer incidence specifically have yielded more conflicting results. Three cohort studies and two case-control studies found no decrease in the risk of endometrial cancer in metformin users compared to nonusers (34, 42, 43, 45, 46). However, these studies show considerable heterogeneity in factors such as study size, indication for metformin use, and duration and method of measurement of metformin exposure (e.g., prescriptions vs. self-report). Notably, a large study of 478,921 Taiwanese women with diabetes showed a significantly decreased incidence of endometrial cancer (hazard ratio [HR] 0.675, 95% CI 0.614–0.742) in metformin users compared to never users (44). When stratified by duration of use or cumulative doses, the decrease in incidence demonstrated a dose-response effect. Additionally, a meta-analysis by Tang and colleagues found that metformin use was associated with a decreased risk of endometrial cancer incidence (RR 0.87, 95% CI 0.80–0.95) (54).

**Table 1:** Studies of metformin use and incidence of endometrial cancer.

**References**	**Design**	**Results**
(42) (UK)	Case-control 2,554 cases, 15,324 controls	Ever-use of metformin not associated with risk of endometrial cancer (OR 0.86, 95% CI 0.63–1.18) Long-term use of metformin (>25 prescriptions) not associated with risk of endometrial cancer (OR 0.79, 95% CI 0.54–1.17)
(34) (USA)	Retrospective cohort 88,107 postmenopausal women (age 50–79)	Self-reported metformin use at study baseline not associated with risk of endometrial cancer (HR 1.64, 95% CI 0.92–2.91)
(43) (USA)	Retrospective cohort 541,128 women (new prescription of metformin or sulfonylurea, any indication)	Metformin use not associated with endometrial cancer risk compared to sulfonylurea use (HR 1.09, 95% CI 0.88–1.35)
(44) (Taiwan)	Retrospective cohort 478,921 women (new diagnosis of type 2 diabetes)	Ever-use of metformin associated with decreased incidence of endometrial cancer (HR 0.675, 95% CI 0.614–0.742) Dose-response was observed when adjusted for duration of metformin use or cumulative metformin doses
(45) (Italy)	Case-control 376 cases, 7,485 controls	Ever-use of metformin use not associated with endometrial cancer risk (OR 1.07, 95% CI 0.82–1.41)
(46) (Finland)	Retrospective cohort 92,366 women (new diagnosis of type 2 diabetes) Nested case-control 590 cases (endometrioid), 11,792 controls	Metformin ever-use associated with increased risk of endometrial cancer in full cohort (OR 1.23, 95% CI 1.03–1.48). Metformin use associated with increased risk of endometrial cancer in nested case-control (OR 1.24, 95% CI 1.02–1.51)

*OR, odds ratio; CI, confidence interval; HR, hazard ratio*.

Other associative studies have focused instead on the relationship between metformin exposure and endometrial cancer outcomes (Table 2). Metformin use in diabetic patients with endometrial cancer was associated with improved overall survival compared to those not taking metformin in two separate studies, including one involving patients with stage III–IV or recurrent endometrial cancer receiving chemotherapy (56, 59). The study by Ko also found improved recurrence-free survival in patients taking metformin. In contrast, some did not find any effect of metformin exposure on survival parameters (57, 58, 61). Still others have reported effects only on certain subgroups of patients. For example, Nevadunsky found increased survival for metformin users only among patients with non-endometrioid but not endometrioid forms of endometrial cancer (55), while Hall reported a significantly lower recurrence rate of only endometrioid endometrial cancers among metformin users (60). As with the incidence research, these studies are limited by heterogeneity and sample size. However, a 2017 meta-analysis including 6 of the above studies supports a higher overall survival rate in metformin-users with endometrial cancer compared to non-metformin users and non-diabetic patients (HR 0.82, 95% CI 0.70–0.95, *I*^2^ = 40%) (62). Finally, a meta-analysis of 28 studies reported that metformin use was associated with decreased all-cause mortality in patients with concurrent diabetes for several cancer types, including endometrial (RR 0.49, 95% CI 0.32, 0.73, *p* < 0.001) (63).

**Table 2:** Observational studies of metformin exposure in endometrial cancer.

	**Number**				
		**DM**		**No DM**	
**References**	**Total**	**MFM**	**No MFM**	**No MFM**	**Results**
(55) (USA)	985	114	136	735	Non-endometrioid endometrioid cancer patients had greater OS in metformin vs. non-metformin users (HR 0.54, 95% CI 0.30–0.97) No association between metformin use and endometrioid endometrial cancer (HR 0.79, 95% CI 0.31–2.0)
(56) (USA)	1,495	196	167	1,132	RFS 1.8 times worse in patients not on metformin compared to metformin users (95% CI 1.1–2.9) OS 2.3 times worse in patients not on metformin compared to metformin users (95% CI 1.3–4.2) TTR not associated with metformin use (HR 1.12, 95% CI 0.6–2.2)
(57) (Poland)	107	30	38	39	No difference in OS between metformin users vs. non-users (*p* = 0.86)
(58) (USA)	1,303	116	161	1,026	OS not significantly different between diabetic metformin-users and diabetic non-metformin users (HR 0.61; 95% CI 0.30–1.23) or non-diabetic patients (HR 1.03; 95% CI 0.57–1.85). PFS not significantly different between diabetic metformin-users compared to diabetic non-metformin users (HR 1.06; 95% CI 0.34–3.30) or non-diabetic patients (HR 1.14; 95% CI 0.46–2.62)
(59) (USA)	349	31	27	291	OS greater in diabetic metformin-users compared to diabetic patients not taking metformin (HR 0.42, 95% CI 0.23–0.78) but not compared to non-diabetic patients (HR 0.65, 95% CI 0.41–1.05)
(60) (USA)	351	64	287		Recurrence rate for all metformin-users vs. non-metformin users not statistically different, but recurrence of type I endometrial cancers was significantly lower for metformin users (1.9%) compared to non-metformin users (10.3%), *p* = 0.05
(61) (Austria)	465	46	41	378	Metformin use not associated with OS (HR 0.9, 95% CI 0.69–1.2) or RFS (HR 1.2, 95% CI 0.8–1.70)

*MFM, metformin; OS, overall survival; HR, hazard ratio; CI, confidence interval; RFS, recurrence-free survival; TTR, time to regression; PFS, progression-free survival*.

## Cellular and molecular mechanisms of metformin inhibition of endometrial cancer

Metabolic alterations in endometrial cancer have been described not only on a systemic but also on a cellular and molecular level. For example, Byrne and colleagues examined microarray data from women with women with type I endometrial cancer and demonstrated that tumor-derived endometrium showed enrichment of genes related to glycolysis and lipogenesis compared to normal endometrium (64). They also reported that multiple human endometrial cancer cell lines showed strong upregulation of the glucose transporter GLUT6 as well as activation of AKT compared to nonmalignant cells. *In vitro* metabolic profiling demonstrated that these changes were associated with upregulation of glycolysis, decreased glucose oxidation, and increased *de novo* lipogenesis. Finally, the authors demonstrated that endometrial cancer cell cultures experience cytotoxicity when exposed to a variety of inhibitors targeting metabolic pathways, including the glycolysis inhibitors 2-deoxy-D-glucose [2-DG] and 3-bromopyruvate (BrPA), the lipogenesis inhibitor 5-(tetradecyloxy)-2-furoic acid (TOFA), the fatty acid oxidation inhibitor etomixir, and the pleiotropic metabolic inhibitor metformin. Further support for the importance of glucose metabolism on endometrial cancer cell growth comes from Han and colleagues, who studied the growth of two endometrial cancer cell lines (ECC-1 and Ishikawa cells) under low, normal, or high glucose conditions (65). High glucose conditions (corresponding to physiologic hyperglycemia) led to increased cell proliferation, *in vitro colony* formation, and increased expression of the GLUT1 glucose transporter along with increased glucose uptake. High glucose also increased phosphorylation of lactate dehydrogenase A (LDHA) and decreased levels of pyruvate dehydrogenase (PDH), suggesting an increase in glycolytic activity. Conversely, low glucose conditions led to increased cell apoptosis, cell cycle arrest, decreased adhesion, and invasion. All of these data support the idea that the metabolic vulnerabilities of endometrial cancer may make it susceptible to therapies such as metformin.

Multiple studies have demonstrated the ability of metformin to inhibit proliferation of both type I and type II human endometrial cancer cell lines in culture (66–73). Metformin treatment of endometrial cancer cell lines upregulates markers of cell cycle arrest (66, 68, 69, 74), apoptosis (66, 67, 69, 72, 73, 75, 76), and autophagy (69, 77), while decreasing markers associated with senescence (66, 74) and inhibiting cell migration (68, 71, 76). The anticancer effects of metformin treatment may not be limited to direct effects on endometrial cancer cells, but may also result from changes to the systemic milieu. Polycystic ovary syndrome (PCOS) is a condition which is associated with endometrial hyperplasia and predisposition to endometrial cancer (78). Endometrial cancer cell lines incubated with sera from PCOS patients showed increased migration and markers of invasiveness such as activity of matrix metalloproteinases (MMP)−2 and −9 compared to cells incubated with sera from healthy controls. In contrast, sera from PCOS patients treated with metformin for 6 months showed attenuation of this effect, with decreased migration and MMP-2/9 activity compared to cells treated with sera from PCOS patients not on metformin (79).

**Figure 1:**
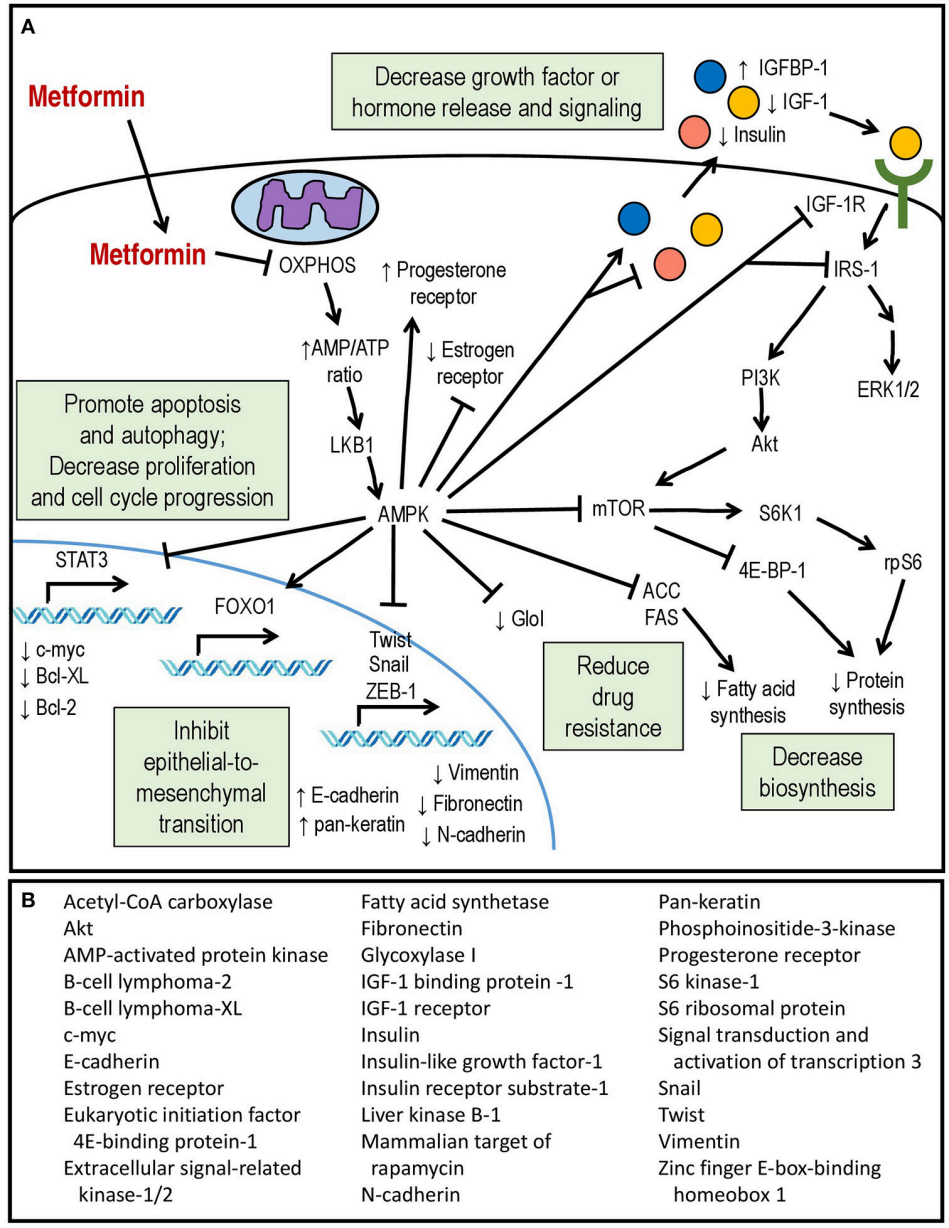
**(A)** Mechanisms of action of metformin within the endometrial cancer cell. **(B)** Downstream molecular targets of metformin showing differential expression or activity in endometrial cancer.

The molecular mechanisms of metformin's effects in endometrial cancer cells are diverse and continue to be an active area of investigation (Figure 1). Its general mechanisms are complex and multifactorial and are reviewed in detail elsewhere (80). Multiple groups have demonstrated that metformin's ability to inhibit oxidative phosphorylation (OXPHOS) at the mitochondrial level is an important mediator of its biologic activity (81, 82). The end result is a decrease in proton gradient across the inner mitochondrial membrane, ultimately leading to reduction in proton-driven synthesis of adenosine triphosphate (ATP) and an increase in the ratio of cellular adenosine monophosphate (AMP) to ATP, caused by imbalance in the rate of ATP production vs. consumption. The decrease in ATP is theorized to be responsible for a key effect of metformin treatment, namely, phosphorylation and activation of the serine/threonine AMP-activated protein kinase (AMPK), a regulatory protein which plays a role in sensing and energy status of the cell and regulating cellular function under conditions of energy restriction (83, 84). This leads to AMP binding to AMPK and a conformational change that allows for phosphorylation/activation of AMPK by liver kinase B1 (LKB1) (85). Activation of AMPK switches cells to a catabolic state via AMPK-mediated phosphorylation and inhibition of key enzymes and transcription factors involved in ATP-consuming synthetic pathways (e.g., glucose, lipid and protein).

Among the known downstream effects of AMPK activation is decreased protein synthesis due to inhibition of the mTOR pathway, resulting in the inhibition of translation by the eukaryotic initiation factor 4E-binding protein-1 [4E-BP1] complex and decreased activity of the S6 kinase 1 (S6K1) responsible for phosphorylation of the ribosomal S6 protein (rpS6) (86, 87). Metformin inhibition of mTOR signaling in endometrial cancer cells has been confirmed by multiple groups (66, 74, 88–90). Other well-described effects of AMPK activation include phosphorylation and inactivation of Acetyl-CoA carboxylase (ACC) leading to downregulation of fatty acid synthesis (83, 91) as well as inhibition of signaling via the insulin-like growth factor-1 receptor (IGF-1R). Metformin inhibition of ACC has not been reported in endometrial cancer cell lines, but Wallbillich did observe decreased expression of fatty acid synthetase (FAS) in metformin-treated tumor tissue from a xenograft model (73). In endometrial cancer cell cultures, metformin treatment lowers secretion of insulin-like growth factor (IGF-1) (70), downregulates expression of insulin receptor (68) and IGF-1R (70, 75), inhibits phosphorylation of IGF-1R (68), and increases expression of insulin-like growth factor-binding protein 1 (IGFBP-1) (75). In other cancer types, this is associated with inhibitory phosphorylation of the signaling adapter, insulin receptor substrate-1 (IRS-1) and inhibition of the downstream PI3K/Akt/mTOR and mitogen-activated protein-kinase/extracellular signal-regulated kinase (MAPK/ERK) pathways (92–94). Inhibition of PI3K/Akt signaling has been observed in metformin-treated endometrial cancer cells (70), and both Akt and ERK1/2 are inhibited in endometrial cancer cells incubated with serum from women with PCOS who are receiving metformin treatment (79). The cumulative result is inhibition of individual cell growth and proliferation, decreased synthesis of proteins and fatty acids, as well as decreased paracrine and endocrine release of pro-proliferative systemic factors.

In addition to IGF-1-related signaling pathways, metformin treatment of endometrial cancer cells has been reported to affect the activity of the transcription factor signal transducer and activator of transcription 3 (STAT3), which is usually activated via signaling by various growth factors and cytokines to dimerize, translocate to the nucleus, and induce transcription of multiple pro-survival and pro-proliferative genes (95). STAT3 levels are elevated in endometrial cancer cells, in particular the serine-phosphorylated form, phospho-STAT3 Ser727 (96). High glucose concentrations induces transcription of STAT3 as well as its upstream regulators Janus kinases 1 and 2 (JAK1/2), while metformin treatment reduces total STAT3 protein as well as phospho-STAT3 Ser727 (73). This is associated with significantly decreased expression of multiple pro-survival downstream targets of STAT3, including c-Myc and B-cell lymphoma (Bcl)-2 and –XL, providing another possible mechanism for metformin's anti-cancer activity. The authors also examined the effect of metformin in a xenograft model of endometrioid endometrial cancer cells. While not statistically significant, metformin exposure was associated with a trend toward decreased tumor size, and analysis of tumor tissues demonstrated decreased expression of STAT3 and its targets (73).

Another recently-reported target of metformin in endometrial cancer is the transcription factor forkhead box protein 1 (FOXO1), which plays numerous roles in cellular function, including regulation of gluconeogenesis, adipogenesis, protection from oxidative stress, and tumor suppression (97). FOXO1 is negatively regulated via inhibitory phosphorylation by Akt, which causes it to translocate out of the nucleus to the cytoplasm where it is degraded (98). Conversely, AMPK activation leads to FOXO1 nuclear localization and activation (99). Zou demonstrated that endometrial cancer cells show decreased levels of phospho-AMPK and total FOXO1 protein, and endometrial cancer tissues show significantly less staining of activated AMPK and higher levels of phospho-Akt compared to controls (72). This is associated with a shift toward cytoplasmic (inactive) rather than nuclear (active) FOXO1 staining. *In vitro*, they reported that metformin treatment increased FOXO1 protein levels, decreased inhibitory FOXO1 phosphorylation, and increased FOXO1 nuclear accumulation in an AMPK-dependent manner, resulting in inhibition of endometrial cancer cell proliferation. Conversely, knockdown of FOXO1 expression using siRNA partially attenuates the antiproliferative effect of metformin on endometrial cancer cells. Metformin treatment inhibited growth of tumors in a xenograft mouse model, and tumor staining also showed increased phospho-AMPK and nuclear localization of FOXO1 as well as decreased staining of the proliferative marker Ki-67 (72). Decreased FOXO1 expression during metformin treatment has been reported by others as well (68).

Metformin treatment has also been shown to inhibit epithelial-to-mesenchymal transition (EMT) in endometrial cancer cell lines. Metformin increases epithelial markers such as E-cadherin and pan-keratin in endometrial cancer cells *in vitro* (76, 100) and decreases mesenchymal markers (e.g., N-cadherin, fibronectin, vimentin) (76, 100) and transcriptional drivers of EMT (e.g., Twist-1, snail-1, zinc finger E-box-binding homeobox-1 [ZEB-1]) (100). In cultured cells, metformin was able to attenuate the molecular and morphologic changes induced by EMT-inducing stimuli such as 17β-estradiol and transforming-growth factor-β (TGF-β) (76). Correspondingly, histologic staining for E-cadherin was significantly higher in endometrial carcinomas taken from patients with a history of metformin use (100).

Metformin has demonstrated the ability to synergize with other endometrial cancer therapies *in vitro*, leading to enhanced apoptosis of cultured endometrial cell lines in the presence of paclitaxel (74, 101), cisplatin (101, 102), or progestin (89). For the latter two, this effect is dependent on downregulation of glycoxylase I (GloI), a mediator of chemotherapy resistance (89, 101). Metformin treatment was also able to increase expression of the progesterone receptor in endometrial cancer cells (88) and sensitize progestin-resistant endometrial carcinoma cells to medroxyprogesterone-induced apoptosis (89). Conversely, metformin alters expression of the estrogen receptor (ER) in endometrial cancer cells, decreasing the ERα isoform while increasing expression of ERβ, with overall inhibition of estradiol-induced proliferation (90). In endometrial cancer patients with type 2 diabetes, metformin leads to decreased expression of the estrogen receptor in tumor tissue compared to insulin treatment (103).

Possible mutation-specific effects of metformin have also been explored in preclinical *in vivo* models of endometrial cancer. Oral metformin is capable of reducing *in vitro* cell proliferation as well as tumor size in xenograft models of multiple human and mouse endometrial cancer cell lines. (67). This effect occurred only in cell lines with activating *K-Ras* mutations but not wild-type *K-Ras* and could be partially attenuated by siRNA-based inhibition of K-Ras expression. Moreover, metformin treatment was shown to cause mislocalization of K-Ras to the cytoplasm in a protein kinase C (PKC)-dependent manner. No association was seen between metformin-responsiveness and *PTEN* mutations. Another study utilized a primary endometrioid endometrial carcinoma xenograft model using cells taken directly from patient biopsies for culture and inoculation into nude mice. The authors noted that one sample contained a *K-Ras* mutation while the other was wild-type; neither tumor was inhibited by metformin treatment, either alone or in combination with cisplatin (104). It should be noted that the dosage of metformin used in this study was lower than in the study by Iglesias (250 mg/kg/day for 21 days vs. 1 g/kg/day for 29-64 days). However, in both these studies, the tumors that displayed no susceptibility to metformin treatment also showed no changes in levels of activated AMPK or downstream mediators, emphasizing the likely importance of this pathway for mediating metformin-induced tumor suppression. Several studies have now shown that higher doses of metformin are required in mice to have antitumor effects than those administered in clinical trials and this may be a reflection of pharmacokinetic differences between rodents and humans (86, 105, 106).

Attention is now also being paid to the impact of metformin on other cellular components of the tumor microenvironment (TME) beyond the cancer cells themselves as described in Rivadeneira and Delgoffe (107). Metformin is capable of decreasing the rate of tumor cell oxygen consumption and thus is able to reduce hypoxia levels within the tumor. The reduction of hypoxia can enhance the activity of agents aimed at stimulating anti-tumor T-cells (108). Further effects of metformin on the immune microenvironment are hypothesized to be mediated through tumor-associated macrophage reprogramming from an M2 to M1-like phenotype (109). A review on the effects of metformin on other tumor cells is beyond the scope of this work. In sum, the pre-clinical data provide sufficient evidence for continued evaluation of metformin's antineoplastic potential. Nonetheless, they also highlight the likely complex interaction between tumor- and patient-specific factors dictating the efficacy of metformin as an anticancer treatment and underscore the need for ongoing human studies.

## Metformin in presurgical and other clinical trials in endometrial cancer

The preponderance of preclinical data has prompted several early phase clinical trials of metformin in human endometrial cancers (Table 3). Many groups have utilized the pre-surgical window approach, in which patients with a biopsy-based histologic diagnosis of endometrial cancer receive metformin treatment during the period prior to hysterectomy. Compared to baseline levels or control patients, patients receiving metformin (between 850 and 2,250 mg daily) showed a post-treatment reduction in markers of DNA replication (topoisomerase IIα) (112) and cellular proliferation (Ki-67) (111, 114, 115, 119). No change in Ki-67 staining was observed by Soliman and colleagues, though notably the dose and duration of metformin exposure was the lowest among these studies (117). Metformin treatment was associated with histologic evidence for inhibition of key signaling pathways including PI3K/Akt/mTOR (111, 112, 114, 116, 117, 119) and MAPK/ERK (112, 117). Tumor immunohistochemistry in one trial also revealed decreased expression of estrogen receptor after metformin treatment (114). Plasma measures of a hyperinsulinemic state, including insulin, IGF-1, glucose, and leptin were significantly reduced post-metformin (111, 112, 116, 117). Furthermore, serum from metformin-treated patients showed decreased ability to stimulate DNA synthesis in cultured endometrial cancer cells (112), suggesting that systemic effects of metformin also play a role in its antiproliferative activity.

**Table 3:** Clinical trials of metformin in endometrial cancer.

**References**	**Design**	**Assessment**	**Treatment**	**No MFM**	**MFM**	**Results**
(110) (Iran)	Non-blinded randomized controlled trial	Endometrial histology after metformin vs. progesterone treatment for dysfunctional uterine bleeding	Metformin 500 mg twice daily or megestrol 40 mg daily for 3 months	21	22	Metformin induced endometrial atrophy in 95.5% (21/22) of patients (including 2 with low grade EEC) compared to 61.9% (13/21) receiving megestrol
(111) (Canada)	Single arm	Effect of metformin on serum and tumor biomarkers during window from biopsy-proven EC diagnosis to resection in non-diabetic patients	Metformin 500 mg three times daily from enrollment to surgery (21–50 days, mean 36.6 days, median 38 days)	10 (banked tissues)	11 (8 EEC, 3 NEEC)	Metformin reduced plasma insulin, IGF-1 and IGFBP-7 and decreased tumor Ki-67 and phospho-rpS6. Metformin treatment led to non-significant increase in plasma IGFBP-1
(112) (Japan)	Single arm	Effect of metformin on serum and tumor biomarkers during window from biopsy-proven EC diagnosis to surgical resection in non-diabetic patients	Metformin starting dose 750 mg daily, increased weekly as tolerated to 1,500–2,250 mg daily (divided) from enrollment to surgery (4–6 weeks)	10 (banked tissues)	31	Metformin reduced tumor topoisomerase IIα, Ki-67, phospho-rpS6, and phospho-ERK1/2 and increased tumor phospho-AMPK Metformin decreased serum insulin, glucose, and IGF-1 metformin decreased ability of sera to stimulate DNA synthesis in cultured cells
(113) (China)	Single arm	Effect of metformin plus estrogen/progesterone combination on early stage EC (Ia) in women with PCOS	Cyproterone acetate 2 mg daily, ethinyl estradiol 35 μg daily, and metformin 1,000 mg daily for 6 months	N/A	5	Estrogen/progesterone treatment combined with metformin led to reversion to normal endometrial epithelium in all patients
(114) (USA)	Single arm	Effect of metformin on tumor biomarkers during window from biopsy-proven EEC diagnosis to surgical resection in obese women	Metformin 850 mg daily from enrollment to surgery (7–28 days, mean 14.65 days)	N/A	20	Metformin decreased tumor Ki-67, phospho-AMPK, phospho-Akt, phospho-rpS6, phospho-4E-BP-1 and ER but did not change PR level Responders had increased serum free fatty acids and tumor staining for markers of fatty acid oxidation and glycogen synthesis
(115) (UK)	Non-randomized controlled trial	Effect of metformin on tumor biomarkers during window from biopsy-proven diagnosis of AEH or EEC to surgical resection	Metformin 850 mg twice daily from enrollment to surgery (7–34 days, median 20 days) vs. no treatment	12 (2 AEH, 10 EEC)	28 (0 AEH, 28 EEC)	Metformin decreased tumor Ki-67 and phospho-4E-BP1 but did not change levels of phospho-Akt, phospho-ACC, phospho-rpS6, ER, PR, or caspase-3
(116) (China)	Non-randomized controlled trial	Effect of metformin on serum and tumor biomarkers during window from biopsy-proven diagnosis of EC and surgical resection in non-diabetic women	Metformin 500 mg three times daily from enrollment to surgery (3–4 weeks) vs. no treatment	30	30	EC patients had higher serum IGF-1, lower tumor phospho-AMPK, and higher tumor phospho-mTOR at baseline than non-EC patients Metformin led to lower serum IGF-1, higher tumor phospho-AMPK and lower tumor phospho-mTOR in EC patients
(117) (USA)	Single arm	Effect of metformin on serum and tumor biomarkers during window from biopsy-proven diagnosis of EC to surgical resection	Metformin 850 mg daily from enrollment to surgery (7–24 days, median 9.5 days)	N/A	20	Metformin decreased serum IGF-1, omentin, insulin, C-peptide, and leptin Metformin decreased tumor phospho-Akt, phospho-rpS6, phospho-ERK1/2 but did not change levels of Ki-67, phospho-ACC or caspase-3
(118) (Japan)	Single arm	Efficacy of metformin in preventing recurrence after progestin therapy for AEH or early stage EC (stage Ia)	Metformin starting dose 750 mg daily, increased weekly as tolerated to 2,250 mg daily (divided, concurrent with medroxyprogesterone acetate-based protocol for 24–36 weeks, continued alone in complete responders until conception or recurrence)	N/A	16 AEH, 13 EC	Metformin maintenance associated with 3-year recurrence-free survival of 89%, compared to expected baseline of 52% recurrence-free survival at 2 years
(119) (China)	Non-randomized controlled trial	Effect of metformin on tumor biomarkers during window from biopsy-proven diagnosis of EC to surgical resection	Metformin 500 mg three times daily for 3–4 weeks	32	33	EC patients had higher tumor Ki-67, PI3K, phospho-Akt, phospho-S6K1, and phospho-4E-BP1 at baseline than non-EC patients Metformin decreased tumor Ki-67, PI3K, phospho-Akt, phospho-S6K1, and phospho-4E-BP1 in EC patients

*MFM, metformin; EC, endometrial cancer; EEC, endometrioid endometrial cancer; NEEC, non-endometrioid endometrial cancer; IGF-1, insulin-like growth factor-1; IGFBP-1/7, insulin-like growth factor binding protein-1/7; rpS6, ribosomal protein S6; ERK1/2, extracellular signal-regulated kinase-1/2; AMPK, AMP-activated kinase; PCOS, polycystic ovary syndrome; Akt, protein kinase B; 4E-BP1, eukaryotic initiation factor 4E-binding protein-1; ER, estrogen receptor; PR, progesterone receptor; AEH, atypical endometrial hyperplasia; ACC, acetyl-CoA carboxylase; mTOR, mammalian target of rapamycin; PI3K, phosphoinositide 3-kinase; S6K1, S6 kinase 1*.

In terms of clinical outcomes, a randomized study by Tabrizi examined the ability of metformin to reverse endometrial hyperplasia or disordered proliferative endometrium in patients with abnormal uterine bleeding compared to megestrol. Metformin was able to induce endometrial atrophy/restore endometrial histology in 95.5% of patients compared to 61.9% in the megestrol group. Significantly, this study included two patients with low grade endometrial carcinomas (stage Ia) who received metformin. After 3 months of treatment, repeat biopsy showed conversion to atrophic endometrium (110). A study of 5 PCOS patients with early stage endometrial carcinoma showed that co-treatment with the oral contraceptive Diane-35 (cyproterone and ethinyl estradiol) and metformin for 6 months led to reversion to normal epithelia on repeat biopsy in all patients (113). This included three patients who had previously been on megestrol treatment for 3 months with documented progesterone resistance. Mitsuhashi also used a combination approach of metformin with medroxyprogesterone to induce remission in patients with atypical endometrial hyperplasia (AEH) or early stage endometrial cancer, and further studied the use of maintenance metformin to prevent relapse. Metformin treatment led to a relapse-free survival of 89% at 3 years, which was higher than their projected baseline of 52% (118). These studies were also notable for their stated goal of fertility preservation, an important consideration for some endometrial cancer patients.

Larger trials are underway, including a phase 3 trial of metformin monotherapy as chemoprevention for endometrial cancer compared to placebo and lifestyle interventions in non-diabetic obese women (NCT01697566) and a phase 2/3 trial by the Gynecologic Oncology Group for advanced (stage III, IVA, IVB) or recurrent endometrial cancer that will compare the addition of metformin vs. placebo to combination paclitaxel/carboplatin as first-line therapy (NCT02065687). Several phase 2 or earlier studies will utilize metformin in combination with hormonal treatments such as megestrol acetate (NCT01968317) or levonorgestrel (as an intrauterine device) for early stage endometrial cancer or complex atypical hyperplasia in young women with the goal of fertility preservation or contraindications to surgery (NCT02990728, NCT02035787, NCT01686126). Also ongoing are a phase 2 study of metformin combined with letrozole and everolimus for advanced or recurrent endometrial cancer (NCT01797523) and a phase 1/2 study of metformin plus metronomic cyclophosphamide and olaparib for advanced or recurrent endometrial cancer (NCT02755844). These efforts will give valuable insight into the preventative and therapeutic value of metformin in endometrial malignancy.

## The metabolic microenvironment as a therapeutic target in endometrial cancer

The role of glucose metabolism in endometrial cancer is also still being explored. Malignant tissues in general have been known to have high levels of glycolytic metabolism, even in the presence of oxygen, a phenomenon known as the Warburg effect after its discoverer, Otto Warburg (120). One theory was that cancer cells are less dependent on oxidative phosphorylation, allowing them to survive in the often relatively hypoxic tumor microenvironment. However, in recent years, a more nuanced model has emerged for some tumor types, known as the reverse Warburg theory (121). This is based on the recognition of heterogeneity in tumor composition; malignant tissues are composed of cancer cells surrounded by diverse types of stromal cells and adipocytes, each of which make contributions to the tumor microenvironment (122, 123). Our research in breast and prostate cancer cell models among others have demonstrated that glycolysis occurs not in the tumor cells themselves, but in the surrounding stromal cells (124–127). This relationship is thought to occur via oxidative stress in the cancer associated stroma (CAS), driven by tumor cell generation of reactive oxygen species and stromal cell loss of caveolin-1 (CAV1), an inhibitor of nitric oxide production (124–129). CAS cells undergo metabolic reprogramming associated with mitophagy (selective degradation of mitochondria by autophagy) and cell autophagy, and they generate high levels of energy-rich metabolites (including lactate and ketones) through glycolysis, which is then shuttled to cancer cells as substrates for oxidative phosphorylation. We have reported that lactate shuttling to tumor epithelial cells is dependent on transporters of the monocarboxylate transporter (MCT) family, with MCT4 being important for lactate efflux from stromal cells and MCT1 for lactate uptake in tumor epithelial cells in a breast cancer cell co-culture system (130). Others have observed similar lactate shuttling in prostate cancer and sarcoma models and have reported that lactate upload promotes tumor cell proliferation and angiogenesis (131, 132). Aside from lactate, Sousa and colleagues have described a similar two-compartment metabolic system in pancreatic ductal adenocarcinoma in which stroma-associated pancreatic stellate cells are stimulated by contact with pancreatic cancer cells to undergo autophagy and secrete primarily alanine which fuels the tricarboxylic acid (TCA) cycle and biosynthesis in the cancer cells themselves (133).

In this model, metformin treatment may play an important part in disrupting cancer cell metabolism via its direct inhibition of mitochondrial respiration in the cancer cells. In agreement with this, our previous results suggest that metformin treatment of cultured breast cancer cells inhibits their ability to induce loss of CAV1 (a marker of tumor-stromal metabolic coupling) in co-cultured fibroblasts (126). We have also observed that a short course of metformin was also able to increase stromal CAV1 expression *in vivo* in patients with head and neck squamous cell carcinoma in a presurgical window of opportunity trial (106).

The existence of such symbiotic metabolic reprogramming has not yet been investigated closely in endometrial cancer, but supportive evidence comes from a study by Latif et al. that showed differential histologic localization for MCT1 (tumor) vs. MCT4 (stroma) for some though not all endometrial cancer samples analyzed (134). High MCT1 expression was also a poor prognostic indicator for recurrence-free, cancer-free, and overall survival in the same study. Moreover, Zhao studied endometrial stromal-epithelial cell interactions in a non-cancer primary cell culture model and showed that epithelial cell proliferation and migration was enhanced when cultured with conditioned media from CAV1 depleted stromal cells (135). A phase 2 study currently underway at our institution utilizes a combination of metformin and doxycycline for the treatment of breast and uterine cancers, with outcomes including the measurement of biomarkers of tumor-stromal metabolic compartmentalization, such as stromal CAV1 and MCT4 and tumor MCT1 (NCT02874430). This will provide new insight into the effects of metformin treatment on the metabolic microenvironment in endometrial cancer.

The Reverse Warburg framework also opens up the possibility of multi-targeted therapies that simultaneously act on aberrant glucose metabolism in both cancer and stromal cells, such as metformin combined with inhibitors of glycolysis, autophagy, or transport of lactate and other energetic substrates. Combined metformin and glycolytic inhibitors have been utilized in xenograft models of breast cancer, gastric cancer, and glioblastoma with synergistic inhibition of tumor growth or prolongation of survival occurring at doses where each agent is ineffective alone (136, 137). Such approaches would exploit the vulnerabilities of tumor-stroma metabolic reprogramming that typically allow for cancer cell survival under a variety of energetic conditions. Overall, the metabolic microenvironment of endometrial cancer represents a promising therapeutic target, one which metformin and other biguanides may be uniquely poised to act upon.

## Conclusions

Endometrial cancer is a disease with few effective treatments for advanced and metastatic disease. In addition, the need for fertility-sparing options for patients with early stage disease means there is a need for more primary or adjunctive treatment approaches. A large body of evidence links endometrial cancer incidence to metabolic conditions such as obesity and hyperglycemic states. Increasing rates of the latter has been mirrored by a rise in the former, particularly in developing countries, highlighting the need for a better understanding of the contribution of the metabolic microenvironment to endometrial cancer tumorigenesis. There is substantial evidence that the mechanisms of nutrient utilization and synthesis are significantly dysregulated in malignancy on both an intracellular and intercellular level. Models such as the reverse Warburg effect especially emphasize the importance of considering the interplay between cancer epithelial cells and their surrounding stroma. Dysregulation of metabolic pathways may represent adaptations that facilitate survival and proliferation in some scenarios (e.g., the idea of the parasitic cancer cell), but can also become liabilities for cancer cells particularly in times of nutrient or energy deprivation. Drugs such as metformin may be uniquely poised to exploit these defects, possibly in conjunction with other therapies that target glucose utilization. Indeed, preclinical and early clinical studies have shown promise for metformin as an adjunctive treatment for endometrial cancer, with effects on both cancer-specific as well as patient-specific metrics. Further study is needed to elucidate the role of metformin as a therapy for endometrial cancer, and several clinical trials are underway that will greatly expand our understanding of its potential benefits.

## Author contributions

JJ and UM-O contributed to the conception and design of the manuscript. TL wrote the first draft of the manuscript. UM-O, RS, CK, SR, NR, and JJ contributed to manuscript revision. All authors read and approved the submitted version.

### Conflict of interest statement

The authors declare that the research was conducted in the absence of any commercial or financial relationships that could be construed as a potential conflict of interest.
